# Sex Differences in Left Ventricular Remodeling and Outcomes in Chronic Aortic Regurgitation

**DOI:** 10.3390/jcm9124100

**Published:** 2020-12-18

**Authors:** Andreas A. Kammerlander, Carolina Donà, Christian Nitsche, Matthias Koschutnik, Amna Zafar, Parastou Eslami, Franz Duca, Stefan Aschauer, Robert Schönbauer, Dietrich Beitzke, Christian Loewe, Udo Hoffmann, Cathérine Gebhard, Christian Hengstenberg, Julia Mascherbauer

**Affiliations:** 1 Division of Cardiology, Medical University of Vienna, 1090 Vienna, Austria; andreas.kammerlander@meduniwien.ac.at (A.A.K.); carolina.dona@meduniwien.ac.at (C.D.); christian.nitsche@meduniwien.ac.at (C.N.); matthias.koschutnik@meduniwien.ac.at (M.K.); franz.duca@meduniwien.ac.at (F.D.); stefan.aschauer@meduniwien.ac.at (S.A.); robert.schoenbauer@meduniwien.ac.at (R.S.); catherine.gebhard@meduniwien.ac.at (C.G.); christian.hengstenberg@meduniwien.ac.at (C.H.); 2 Cardiovascular Imaging Research Center, Massachusetts General Hospital, Harvard Medical School, Boston, MA 02114, USA; azafar@mgh.harvard.edu (A.Z.); peslami1@mgh.harvard.edu (P.E.); uhoffmann@mgh.harvard.edu (U.H.); 3 Section of Cardiovascular and Interventional Radiology, Department of Biomedical Imaging and Image-Guided Therapy, Medical University of Vienna, 1090 Vienna, Austria; dietrich.beitzke@meduniwien.ac.at (D.B.); christian.loewe@meduniwien.ac.at (C.L.); 4 Department of Nuclear Medicine, University Hospital Zurich and Center for Molecular Cardiology, University of Zurich, 8091 Zurich, Switzerland

**Keywords:** aortic regurgitation, sex differences, cardiovascular magnetic resonance imaging

## Abstract

Background: Left ventricular (LV) dilatation is a key compensatory feature in patients with chronic aortic regurgitation (AR). However, sex-differences in LV remodeling and outcomes in chronic AR have been poorly investigated so far. Methods: We performed cardiovascular magnetic resonance imaging (CMR) including phase-contrast velocity-encoded imaging for the measurement of regurgitant fraction (RegF) at the sinotubular junction, in consecutive patients with at least mild AR on echocardiography. We assessed LV size (end-diastolic volume indexed to body surface area, LVEDV/BSA) and investigated sex differences between LV remodeling and increasing degrees of AR severity. Cox-regression models were used to test differences in outcomes between men and women using a composite of heart failure hospitalization, unscheduled AR intervention, and cardiovascular death. Results: 270 consecutive patients (59.6% male, 59.8 ± 20.8 y/o, 59.6% with at least moderate AR on echocardiography) were included. On CMR, mean RegF was 18.1 ± 17.9% and a total of 65 (24.1%) had a RegF ≥ 30%. LVEDV/BSA was markedly closer related with AR severity (RegF) in men compared to women. Each 1-SD increase in LVEDV/BSA (mL/m^2^) was associated with a 9.7% increase in RegF in men and 5.9% in women, respectively (*p*-value for sex-interaction < 0.001). Based on previously published reference values, women—in contrast to men—frequently had a normal LV size despite severe AR (e.g., for LVEDV/BSA on CMR: 35.3% versus 8.7%, *p* < 0.001). In a Cox-regression model adjusted for age, LVEDV/BSA and RegF, women were at significantly higher risk for the composite endpoint when compared to men (adj. HR 1.81 (95%CI 1.09–3.03), *p* = 0.022). Conclusion: In patients with chronic AR, LV remodeling is a hallmark feature in men but not in women. Severity of AR may be underdiagnosed in female patients in the absence of LV dilatation. Future studies need to address the dismal prognosis in female patients with chronic AR.

## 1. Introduction

Left ventricular (LV) dilatation is a hallmark feature of patients with chronic aortic regurgitation (AR) [1]. Transthoracic echocardiography (TTE) is the preferred imaging modality for AR severity assessment [2]. Current guidelines suggest an integrated approach for assessment of AR severity using pressure-half-time (PHT), color Doppler jet size, vena contracta (VC) width of the regurgitant jet, proximal isovelocity surface area (PISA) radius, and the presence of holodiastolic retrograde flow in the descending aorta (HRF) [2]. These measurements, however, are strongly influenced by the quality of acoustic windows, flow convergence shape, and machine settings [2,3], resulting in substantial inter-rater variability [4]. In contrast, LV dilatation represents the true hemodynamic sequela of chronic AR, and LV size is relatively easy to assess. While current guidelines recognize LV size as an important feature in assessing the severity of AR, they do not specify a cut-off LV size to define severe AR [2,3].

Cardiovascular magnetic resonance imaging (CMR) allows further AR quantification by assessment of regurgitant volume (RegV) and regurgitant fraction (RegF) using phase-contrast velocity-encoded imaging [5]. In addition, CMR is considered the gold-standard for evaluation of ventricular volumes and function, and dimensions of the thoracic aorta [6]. Current guidelines recommend performing CMR for RegF quantification in cases with inconclusive echocardiographic workup, however, there is no consensus on how to define severe AR by CMR [7,8]. Several cut-off values for RegF have been suggested, ranging from 27–33% [9,10,11].

Previous studies have linked LV dilatation with incident worsening of clinical status [12], and the 2014 ACC/AHA guidelines established criteria for LV size as an indication for surgical intervention. However, it is unknown whether previously published upper limits of normal for LV dilatation correlate with the presence of severe AR. In addition, there is no reported data on sex differences in LV remodeling in the setting of chronic AR and limited data is available on sex differences in clinical outcomes. 

In this study of consecutive patients with chronic AR undergoing a comprehensive diagnostic workup, including echocardiography and CMR with phase-contrast analysis, we investigated sex differences in (A) the relationship of LV remodeling and AR severity; (B) the prevalence of LV dilatation, based on previously published upper limits of normal; and (C) incident heart failure hospitalization, unscheduled AR intervention, and cardiovascular death. 

## 2. Experimental Section

### 2.1. Setting and Study Design

Patients were recruited at a single tertiary referral center with a high-volume multimodality-imaging facility. Consecutive patients with chronic AR were invited to undergo CMR within four weeks of TTE. Both patients with and without a class I indication for aortic valve surgery were eligible for study entry. Preliminary data from this cohort on the diagnostic utility of holo-diastolic retrograde flow in the descending aorta have been published previously [13]. We excluded patients with (1) prosthetic heart valves in aortic position; and (2) conditions affiliated with LV dilatation, including dilated cardiomyopathy, ischemic cardiomyopathy, ventricular septal defects, and severe primary mitral regurgitation. Other valvular heart diseases were not excluded to allow for a real-world scenario of AR assessment. Patients with acute AR or endocarditis were not included in the study. The ethics committee approved the study protocol. All patients provided written informed consent. 

### 2.2. Echocardiography

Board certified cardiologists with extensive expertise in echocardiography performed AR assessment, using high-end scanners, such as GE Vivid S70 (GE Healthcare, Wauwatosa, WI, USA). We used an integrated approach in line with current recommendations [2,3]. In parasternal long-axis views, we measured the regurgitant jet size, its size in relation to the LV outflow tract diameter, and the vena contracta (the narrowest width of the regurgitant jet at the valvular level). Apical five-chamber views were used for assessment of the proximal isovelocity surface area (PISA) as well as the effective regurgitant orifice area (EROA) and RegV, as described previously [2]. Pressure half time (PHT) was measured by continuous wave Doppler. On suprasternal views, HRF in the descending aorta was assessed by pulsed wave Doppler [2]. LV end-diastolic diameters (LVEDD) were measured in apical four-chamber views, while LV end-diastolic and end-systolic volumes (LVEDV, LVESV), and LV ejection fractions (LVEF) were calculated using the biplane Simpson’s method. LV size parameters, were indexed to body surface area, and integrated into the AR quantification process [14,15]. 

### 2.3. Cardiac Magnetic Resonance Imaging

All CMR studies were performed on a dedicated 1.5-T cardiac system (Avanto, Siemens Medical Solutions, Erlangen, Germany) using standard protocols [16]. Phase-contrast velocity-encoded sequences were acquired perpendicular to the ascending aorta at the sinotubular junction. Typical parameters were: Repetition time: 4.8 ms, echo time: 2.8 ms, matrix: 320 × 300 mm, flip angle: 12°, temporal resolution: 25–55 ms, and velocity window: 2.5–4.0 m/s. We measured RegV and RegF at the sinotubular junction, as previously described [13,17]. Dedicated software (cmr42, Circle Cardiovascular Imaging Inc., Calgary, AB, Canada) was used for CMR analyses. A stack of short axis views was used for volumetric assessment of LV end-diastolic and end-systolic volumes (LVEDV, LVESV) and ejection fraction (LVEF). Reads were performed by readers with at least five years of experience in CMR (AK and JM). A semi-automatic approach with manual correction was used for RegV and RegF assessment. CMR investigators were blinded to echocardiographic results. Interobserver variabilities for RegF have been previously reported [13]. For interrater variability of RegV and RegF, 20 randomly chosen patients were read by two investigators (A.K, M.K.) blinded to the other reader’s results.

### 2.4. Follow-Up and Outcomes

Patients were prospectively followed and contacted at a 6-month interval, including on-site visits or telephone contact, respectively. In addition, the nationwide hospital chart system was reviewed to identify the composite of (A) heart failure hospitalization; (B) unscheduled AR intervention in case of clinical worsening equivalent to symptoms of cardiac decompensation; and (C) cardiovascular death. Two study members (AK and JM) served as adjudication committee for each event.

### 2.5. Statistical Analysis

Continuous data are expressed as mean ± SD and categorical variables are presented in percent and/or total numbers. Wilcoxon rank-sum tests and chi-square or Fisher’s exact tests were used for comparison between male and female patients, as appropriate. Linear regression models, adjusted for age, were used to test the association between LV size parameters and RegF as a metric variable, and standardized beta coefficients are reported for better comparison. To compare the strength of association between LV size parameters and severe AR, we compared sex-specific odds ratios, adjusted for age, per 1 SD increase for each parameter. Consistent with previous literature, we defined severe AR as RegF ≥ 30%. Sensitivity analyses were performed for each published cut-off value, namely a RegF ≥ 27% and ≥33% [9,10,11,12]. Diagnostic performance of published upper limits of normal to define LV dilatation was assessed. On echocardiography, these are 58.4 mm LVEDD, 150 mL LVEDV, and 74 mL/m^2^ LVEDV/BSA for men, and 52.2 mm LVEDD, 106 mL LVEDV, and 61 mL/m^2^ LVEDV/BSA for women [14]. On CMR, these are 203 mL LVEDV and 100 mL/m^2^ LVEDV/BSA for men < 60 years, and 190 mL LVEDV and 94 mL/m^2^ LVEDV/BSA for men ≥ 60 years. Corresponding thresholds for women were 174 mL LVEDV and 95 mL/m^2^ LVEDV/BSA (<60 years) and 162 mL LVEDV and 86 mL/m^2^ LVEDV/BSA (≥60 years) [18]. Cox-regression models were used to investigate the impact of LV size and sex on the primary endpoint (heart failure hospitalization and cardiovascular death). Univariable analyses were performed for the most important clinical (age, sex, body mass index, hypertension, atrial fibrillation, diabetes, hyperlipidemia, coronary artery disease, previous stroke, hematocrit, estimated glomerular filtration rate) and imaging variables (bicuspid aortic valve, CMR LVEDV/BSA, CMR LVEF, CMR RVEDV/BSA, CMR RVEF, CMR RegF, CMR RegV). LVEDV/BSA, and RegF results were adjusted for each other and age in sex-specific analyses.

Two different multivariable Cox regression models were run to investigate the impact of sex on clinical outcomes: (A) adjusting for a prespecified set of covariates (age, LVEDV/BSA, and RegF), and (B) adjusting for all abovementioned variables with a significant impact on a univariable level. Scheduled interventions of the aortic valve were not considered as an event and censored at the time of intervention.

In an exploratory step, receiver operating characteristics (ROC) reporting areas under the curve (AUC) based on the Youden index approach were performed to investigate an optimal cut-off for CMR LVEDV/BSA in men and women using the composite endpoint as outcome.

Intraclass correlation coefficients (ICC) report interrater variability. For all statistical tests, the level of significance was set to *p* < 0.05, unless stated otherwise. Statistical analyses were performed using STATA 15.1 (StataCorp, College Station, TX, USA).

## 3. Results

### 3.1. Clinical Data

**Figure 1:**
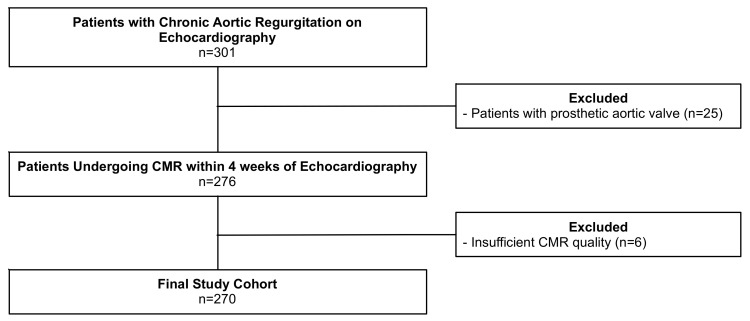
Patient flowchart. CMR indicates cardiovascular magnetic resonance.

Out of 301 patients, we excluded six patients due to poor CMR quality and 25 due to the presence of a prosthetic valve in the aortic position. Hence, the final study cohort consisted of 270 patients (see Figure 1). No patient had a history of or findings consistent with non-ischemic or ischemic cardiomyopathy, ventricular septal defect, or primary mitral regurgitation. 

Baseline characteristics of patients are summarized in Table 1. In brief, mean age was 59.8 ± 20.8 years and 40.4% were female. 55 (20.4%) patients had a bicuspid aortic valve, which was present more often in men compared to women (29.0% versus 7.0%, *p* < 0.001). 47 (17.4%) patients had a class I indication for AVR (14.7% women versus 19.3% men, *p* = 0.221) at the time of echocardiography. 

### 3.2. Echocardiographic Results

On echocardiography [2,3] 83 (30.7%) patients had mild, 99 (36.7%) had moderate, 44 (16.3%) had moderate-to-severe, and 44 (16.3%) severe AR. Baseline imaging variables are displayed in Table 2, stratified by sex. 73 (27.0%) had at least moderate aortic stenosis, of whom 38 (52.1%) had both AR and AS of at least moderate severity. On echocardiography, mean LVEDD was 49.0 ± 9.0 mm, LVEDV and LVEDV/BSA were 134.2 ± 60.2 mL, and 70.1 ± 29.1 mL, respectively. Mean LVEF was 57.4 ± 13.7%. As expected, men had significantly larger LV size parameters compared to women (*p* < 0.001 for all). 

### 3.3. Cardiovascular Magnetic Resonance Imaging Results

Table 2 reports CMR findings at baseline. Mean LVEDV, LVEDV/BSA and LVEF were 181.2 ± 73.6 mL, 94.7 ± 35.3 mL/m^2^, and 52.0 ± 12.4%, respectively. RegF and RegV at the sinotubular junction were 19.6 ± 24.1 and 18.1 ± 18.0, respectively. A total of 24.1% had severe AR, defined as RegF ≥ 30%, which was significantly more frequent in men as compared to women (29.8% versus 15.6%, *p* = 0.01). We found excellent agreement for RegF and RegV between two readers in 20 randomly chosen patients (ICC 0.93 and 0.92, respectively). 

### 3.4. Left Ventricular Size and Regurgitation Fraction 

**Figure 2:**
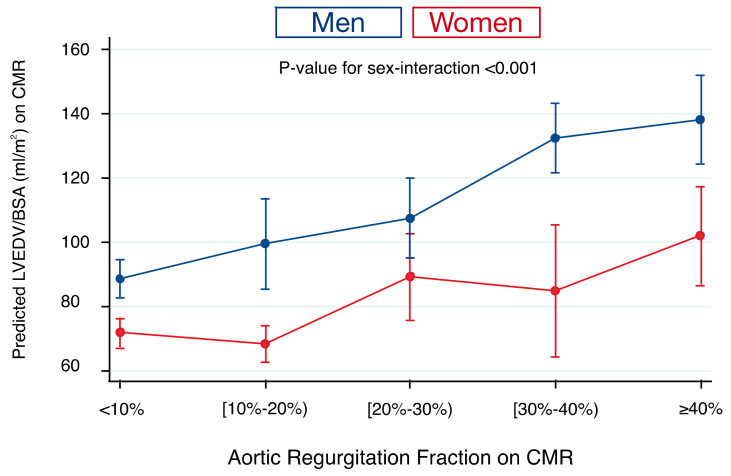
Sex-interaction of the association between left ventricular (LV) end-diastolic volume indexed to body surface area (LVEDV/BSA) and aortic regurgitation (AR) fraction on cardiovascular magnetic resonance imaging (CMR). There is a substantial overlap of LV size across different degrees of AR severity in women but not in men.

LVEDV/BSA on CMR was markedly closer related with AR severity (RegF) in men compared to women. Each 1-SD increase in LVEDV/BSA (mL/m^2^) was associated with a 9.7% increase in RegF in men versus a 5.9% in women (*p*-value for sex-interaction < 0.001). Figure 2 depicts the association of LVEDV/BSA and RegF on CMR. There was a substantial overlap of LV size across different degrees of AR severity in women but not in men (Figure 2). 

**Figure 3:**
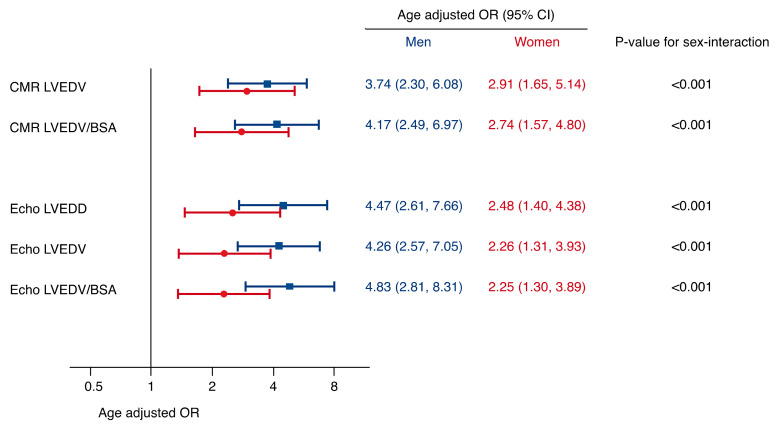
Strengths of association between echocardiographic and cardiovascular magnetic resonance imaging (CMR) parameters of left ventricular (LV) size with presence of severe aortic regurgitation (AR, ≥30% regurgitation fraction on CMR). Sex-specific odds ratios (OR) are adjusted for age and presented per 1-SD increase of each parameter. In men, CMR and echocardiographic parameters showed a similar strength of association with severe AR. In female patients, however, this association was markedly lower compared to male patients, especially on echocardiography (*p*-value for sex-interaction <0.001 for all).

Comparing the different measures of LV size per 1-SD increase, we observed a similar performance between echocardiographic and CMR parameters (Figure 3, Table 3). However, the magnitude of association was markedly lower in women as compared with men across all measures of LV size (*p*-value for sex-interaction < 0.001 for all). 

### 3.5. Association of Severe AR and LV Size

**Figure 4:**
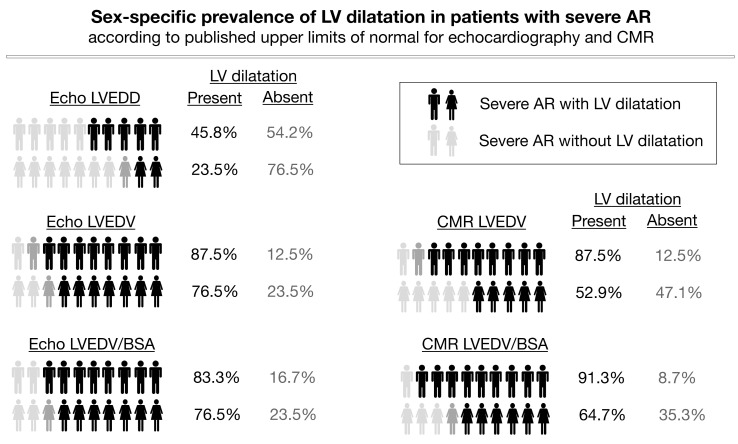
Sex-specific prevalence of left ventricular (LV) dilatation, according to published upper limits of normal, on echocardiography and CMR in patients with severe aortic regurgitation. LV dilatation is a key finding in men but often absent in women.

In the 65 patients with severe AR, here defined as RegF ≥ 30% on CMR, the presence of LV dilatation varied on echocardiography from 40% for LVEDD to 84.6% for LVEDV, and in CMR from 78.5% for LVEDV to 84.6% for LVEDV/BSA. Figure 4 depicts sex-specific differences. LV dilatation was significantly more often present in men, as compared to women, when CMR thresholds for LVEDV and LVEDV/BSA were used (87.5% versus 52.9%, *p* < 0.001, and 91.3% versus 64.7%, *p* < 0.001). Also, echocardiography more often classified male patients as having a dilated LV when using LVEDV or LVEDV/BSA as compared to women, although not statistically significant (87.5% versus 76.5%, *p* = 0.145, and 83.3% versus 76.5%, *p* = 0.063). LVEDD on echocardiography only identified LV dilatation in 45.8% of male patients with severe AR, and in 23.5% in female patients (*p* = 0.028). 

### 3.6. Outcomes

**Figure 5:**
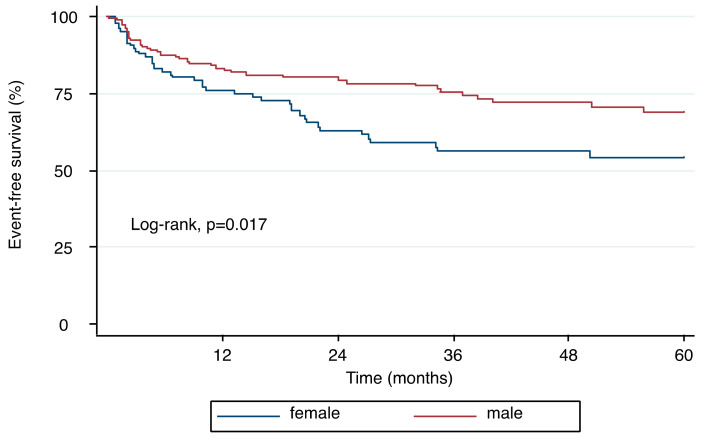
Kaplan–Meier plot demonstrating the relationship between sex and time to heart failure hospitalization and cardiovascular death. Female patients with chronic AR had significantly worse outcomes when compared to men (log-rank, *p* = 0.017).

During a mean follow-up of 32.4 ± 26.5 months a total of 81 events were recorded. By Kaplan–Meier analysis, female patients were significantly more likely to experience the composite endpoint when compared to men (Figure 5, log-rank, *p* = 0.017). Results of the univariable Cox regression are displayed in Table 4. 

By age adjusted Cox regression models, LVEDV/BSA on CMR was significantly associated with a dismal prognosis and women (adj. HR per 1-SD increase 1.44 (95%CI: 1.08–1.91), *p* = 0.014), but not in men (adj. HR per 1-SD increase 1.29 (95%CI: 0.95–1.75), *p* = 0.104), although showing a similar trend. Further adjustment for RegF on CMR attenuated the effect of LVEDV/BSA in both sexes (adj. HR in women per 1-SD increase 1.24 (0.91–1.69), *p* = 0.164 versus 1.24 (0.86–1.77) in men, *p* = 0.246). In that model (including age and LVEDV/BSA), RegF had a trend towards dismal outcomes in both men and women (adj. HR per 1% increase: 1.02 [1.00–1.03], *p* = 0.069 and 1.03 (1.01–1.05), *p* < 0.001). 

In a multivariable Cox-regression model adjusting for the prespecified set of covariates (age, LVEDV/BSA, and RegF on CMR) female sex remained significantly associated with higher risk for events (adj. HR 1.81 (95%CI 1.09–3.03), *p* = 0.022). In a separate analysis adjusting for factors associated with a significant impact on outcome on a univariable level (Table 4), female sex remained significantly associated with higher risk for adverse events (adj. HR 1.33 (95%CI 1.03–2.52), *p* = 0.031) along with RegF (per 1% increase adj. HR 1.02 (1.00–1.03), *p* = 0.033). 

In an exploratory step, ROC analysis revealed an optimal cut-off for CMR LVEDV/BSA of 108 mL/m^2^ in men and 74 mL/m^2^ in women to be associated with outcome, however, demonstrating moderate overall performance (AUC 0.60 and 0.64 respectively). 

All results were consistent when excluding patients with BAV.

## 4. Discussion

In the present study we investigated sex-specific differences in LV dilatation and outcomes in patients with chronic AR. We report three main findings: (1) LV remodeling is more pronounced in men when compared to women; (2) in women LV dilatation is frequently absent when applying standard cut-off values; and (3) women with chronic AR face a more dismal prognosis than men.

### 4.1. Left Ventricular Remodeling in Aortic Regurgitation

Accurate assessment of AR is challenging. Current guidelines recommend using an integrated echocardiography approach based on AR jet size, VC, PISA, EROA, and PHT, as the first step [2,3]. In case of inconclusive echocardiographic AR assessment, a class Ia recommendation is given for CMR in the European recommendations [2]. The 2017 US recommendations, endorsed by the American Society of Echocardiography and the Society for Cardiovascular Magnetic Resonance list specific indications for using CMR for AR assessment, that include suboptimal image quality on echocardiography, discordance between LV dilatation and Doppler assessment of AR severity, and in the presence of at least moderate AR on echocardiography but suboptimal imaging of LV size and function [3]. 

Although LV dilatation is highlighted as a key finding in both recommendations, it is the only parameter in the integrated algorithm without specific cut-off values [2,3]. Several population-based studies have reported age-, sex-, and race- specific cut-offs for the definition of normal LV size for echocardiography and CMR [14,18,19]. These definitions rely on the statistical measures of spread (90th percentile or mean + 2 SD) to define the upper limit of normal in a healthy population. In clinical practice, these thresholds are used to define LV dilatation [20]. However, patients evaluated for AR severity may have different physiologic characteristics, such as arterial hypertension, which may impact LV size when compared to healthy derivation cohorts.

In contrast to lacking thresholds for the definition of LV dilatation in severe AR, specific cut-off values of LV size are implemented as gatekeeper for indication for surgery in both European and American guidelines. Interestingly, recommendations are based on LVEDD and LV end-systolic diameters, not volumes, and sex-specific thresholds are lacking [8,21]. 

In our study, we could demonstrate a clear and linear relationship between increasing RegF on CMR and LV size in men whereas a substantial overlap of LV size is found in women across different degrees of AR severity. Limited data on why LV dilatation is less pronounced in severe AR in women compared to men. In an animal model using induced chronic volume overload, female—in contrast to male—rats did not develop LV dilatation, which is similar to our findings [22]. However, the underlying mechanisms are unknown. Data from patients’ aortic stenosis show similar differences between men and women with women having less pronounced signs of LV remodeling than men [23]. One potential mechanism may be a protective effect of estrogen as demonstrated in pressure overload conditions by inhibiting calcineurin which is able to activate a cascade of transcription factors resulting in adverse cardiac remodeling [24]. Conflicting data, however, come from a large retrospective data in patients with mitral regurgitation, sharing a volume overload condition with AR, where female patients had higher LV dimensions when indexed to BSA [25]. In summary, sex-specific differences in LV remodeling in volume or pressure overload conditions are incompletely understood and should prompt further studies exploring the pathophysiologic mechanisms. 

Also, it is important to note that similar to patients with severe aortic stenosis, in whom left ventricular hypertrophy is not a mandatory finding, patients with severe AR may lack LV dilatation and an integrative approach according to current guidelines should be applied. 

### 4.2. Sex-Specific Outcome Differences in Chronic Aortic Regurgitation

Given the increased prevalence of bicuspid aortic valve and resultant severe AR in males, women have long been under-represented in studies investigating chronic AR [26,27,28,29]. This has been shown to result in higher mortality rates in women compared to men with severe AR [30].

Previous studies have linked LV dilatation with incident worsening of clinical status and established it as an indication for surgery in chronic AR [1,12]. Current recommendations promote LVEDD (>70 mm, [8] >65 mm [21]) and LV end-systolic diameter (>50 mm, [8,21] >25 mm/m^2^ indexed to BSA [8]) as gatekeeper for surgery in asymptomatic patients with severe AR [8,21]. However, several echocardiographic studies challenged these thresholds, especially for women, and suggest that lower thresholds should be used [31].

In a CMR study including 113 patients with moderate and severe AR, Myerson et al. reported thresholds for LV size to predict progression of symptoms and incident indication for surgery. They report an LVEDV of 246 mL or higher and a LVEDV/BSA of 129 mL/m^2^ or higher to be most closely related to the outcome. However, 81% of their study cohort was male, and presence of LV dilatation as well as sex-specific thresholds were not reported [12]. Their thresholds are similar to those of the male patients with severe AR in our cohort. However, female patients have significantly smaller left ventricles, irrespective of indexing LVEDV to BSA. Indeed, the majority of female patients with severe AR in our cohort had a small LV, when compared to thresholds proposed by Myerson’s group [12]. In our study, female sex was associated with a significantly higher hazard of heart failure hospitalization and cardiovascular death even after adjustment for age LV size and RegF. LV size was associated with higher event rates in both sexes but this effect was attenuated when adjusting for RegF. 

A major factor limiting comparison of studies is the lack of data on chronicity of AR. It is known that women present at a later stage with presumably more long-standing disease as compared to men [30]. This may explain our finding, in line with previous reports [30], of a more dismal prognosis in female patients with chronic AR despite less pronounced LV remodeling. 

Of note, we could not define an ideal cut-off value for LV size to predict dismal outcome as reflected by modest AUC results in our cohort. These findings highlight that LV size should not be the single gatekeeper for clinical decision making.

### 4.3. Limitations

Several limitations merit comment. Due to the single-center nature, a center-specific bias cannot be excluded. However, advantages of single center studies include the inclusion of a homogenous patient population, adherence to a constant clinical routine, and standardized CMR and TTE examinations. Also, results of our predominantly Caucasian study population may not address the specific diagnostic needs of other ethnicities. Detailed data on AR chronicity was not available but no patient had acute AR or active endocarditis. In addition, sex-specific differences in the clinical management and delayed diagnosis of valvular heart disease in women prior to the study inclusion are likely and should be taken into account as potential confounder. However, this would not explain the absence of LV dilatation in female patients with severe AR, as observed in our study. Furthermore, our study was not designed to test optimal timing of valve intervention in patients with chronic AR. 

## 5. Conclusions

In summary, there are important sex differences in LV remodeling in chronic AR. Increasing AR severity is strongly associated with increase in LV size in men but less pronounced in women. In women, severe AR may be undiagnosed in the absence of LV dilatation based on established thresholds. Future studies are warranted to address the dismal outcome in female patients with chronic AR. 

## Figures and Tables

**Table 1:** Clinical baseline characteristics, stratified by sex.

	All Patients *n* = 270	Women *n* = 109 (40.4%)	Men *n* = 161 (59.6%)	*p*-Value
Age (years)	60 ± 21	64 ± 20	57.3 ± 21.0	0.01 *
BMI (kg/m^2^)	26.3 ± 4.6	26.5 ± 5.4	26.2 ± 4.1	0.84
BSA (m^2^)	1.90 ± 1.26	1.77 ± 0.21	1.99 ± 0.19	<0.001 *
Hypertension (%)	65.4	68.0	63.3	0.46
Atrial fibrillation (%)	28.1	27.5	28.6	0.85
Diabetes (%)	13.6	18.6	9.5	0.046 *
Hyperlipidemia (%)	39.5	31.7	45.7	0.03 *
CAD (%)	27.5	23.5	30.7	0.23
Previous PCI (%)	10.0	4.9	14.2	0.02 *
Previous CABG (%)	7.9	7.8	7.9	0.99
Previous MI (%)	9.6	5.9	12.6	0.09
Previous stroke (%)	3.9	4.3	3.5	0.75
Hematocrit (g/dL)	39.4 ± 5.1	37.1 ± 3.9	41.0 ± 5.2	<0.001 *
eGFR (mL/min/1.73 m^2^)	66.1 ± 25.9	64.1 ± 26.8	67.5 ± 25.3	0.20
Bicuspid valve (%)	20.5	7.0	29.0	<0.001 *

Numbers indicate % or median and corresponding interquartile range. * indicates statistical significance, defined as a *p*-value < 0.05. BMI indicates body mass index; BSA, body surface area; CAD, coronary artery disease; PCI, percutaneous coronary intervention; CABG, coronary artery bypass grafting; MI, myocardial infarction; eGFR, estimated glomerular filtration rate.

**Table 2:** Echocardiographic and cardiovascular magnetic resonance imaging (CMR) parameters, stratified by sex.

	All Patients (*n* = 270)	Women (*n* = 109, 40.4%)	Men (*n* = 161, 59.6%)	*p*-Value
ECHOCARDIOGRAPHIC PARAMETERS				
LA diameter (mm)	56.5 ± 10.4	55.3 ± 9.5	57.2 ± 11.0	0.24
RA diameter (mm)	55.3 ± 10.0	54.6 ± 9.7	55.8 ± 10.2	0.75
LVEDD (mm)	49.0 ± 9.0	44.3 ± 6.5	52.1 ± 9.1	<0.001 *
LVESD (mm)	41.3 ± 9.7	36.2 ± 7.0	44.6 ± 9.8	<0.001 *
LVEDV (mL)	134.2 ± 60.2	99.5 ± 32.7	157.7 ± 63.2	<0.001 *
LVEDV/BSA (mL/m^2^)	70.1 ± 29.1	56.7 ± 18.5	79.2 ± 31.4	<0.001 *
LVESV (mL)	62.5 ± 37.9	42.6 ± 19.8	75.9 ± 41.3	<0.001 *
LVESV/BSA (mL/m^2^)	32.6 ± 18.9	24.3 ± 11.4	38.2 ± 20.8	<0.001 *
LVEF (%)	55.6 ± 11.8	58.9 ± 10.4	53.3 ± 12.1	<0.001 *
RVEDD (mm)	34.4 ± 7.1	33.7 ± 6.3	34.9 ± 7.5	<0.001 *
RV FAC (%)	44.8 ± 11.0	44.5 ± 11.0	45.1 ± 11.1	0.13
TAPSE (mm)	21.0 ± 6.0	19.7 ± 5.7	21.8 ± 6.1	0.72
IVS (mm)	13.4 ± 3.0	12.8 ± 2.8	13.9 ± 3.1	0.02 *
Peak TR velocity (m/s)	3.1 ± 0.6	3.1 ± 0.6	3.0 ± 0.7	0.002 *
Valvular lesions				
AR ≥ moderate (%)	59.6	51.4	65.2	0.02 *
AS ≥ moderate (%)	27.0	31.2	24.2	0.21
MR ≥ moderate (%) ^‡^	23.3	26.6	21.1	0.30
TR ≥ moderate (%)	21.5	31.2	14.9	0.001 *
CMR PARAMETERS				
LA diameter (mm)	61.6 ± 11.1	60.3 ± 9.4	62.5 ± 12.0	0.31
LA volume (mL)	89.4 ± 48.0	81.8 ± 4.0	94.5 ± 51.5	0.06
RA diameter (mm)	61.6 ± 9.6	59.8 ± 8.2	62.9 ± 10.2	0.01 *
RA volume (mL)	93.6 ± 66.0	75.0 ± 37.9	106.3 ± 77.3	<0.001
IVS (mm)	12.5 ± 3.2	11.8 ± 3.2	12.9 ± 3.2	0.001 *
LV mass (g)	157.7 ± 59.1	128.7 ± 34.7	188.9 ± 64.3	<0.001 *
LV mass/BSA (g/m^2^)	83.7 ± 28.6	72.7 ± 22.2	95.4 ± 30.2	<0.001 *
LVEF (%)	57.4 ± 13.7	62.9 ± 12.8	53.7 ± 13.1	<0.001 *
LVEDV (mL)	181.2 ± 73.6	133.6 ± 38.9	213.5 ± 74.1	<0.001 *
LVEDV/BSA (mL/m^2^)	94.7 ± 35.3	76.1 ± 22.0	107.3 ± 37.0	<0.001 *
LVESV (mL)	82.4 ± 53.3	52.2 ± 31.1	102.8 ± 55.4	<0.001 *
LVESV/BSA (mL/m^2^)	42.9 ± 26.6	29.8 ± 17.5	51.8 ± 28.0	<0.001 *
Cardiac output (L/min)	6.5 ± 2.3	5.5 ± 1.3	7.2 ± 2.5	<0.001 *
RVEF (%)	52.0 ± 12.4	54.3 ± 11.8	50.5 ± 12.6	0.02 *
RVEDV (mL)	155.0 ± 55.1	129.6 ± 40.6	172.3 ± 57.0	<0.001 *
AR quantification parameters				
RegV (mL)	19.6 ± 24.1	9.3 ± 13.8	26.0 ± 26.8	<0.001 *
RegF (%)	18.1 ± 18.0	12.9 ± 15.1	21.6 ± 19.0	<0.001 *
RegF ≥ 27%	27.8	15.6	35.4	<0.001 *
RegF ≥ 30%	24.1	15.6	29.8	0.01 *
RegF ≥ 33%	20.7	13.8	25.5	0.02 *

Numbers indicate % or mean and standard deviation. LA indicates left atrium; RA, right atrium; LV, left ventricle; EDD, end-diastolic diameter; EDV, end-diastolic volume; BSA, body surface area; EF, ejection fraction; RV, right ventricle; FAC, fractional area change; TAPSE, tricuspid annular plane systolic excursion; IVS, interventricular septal thickness; TR, tricuspid regurgitation; MR, mitral regurgitation; AS, aortic stenosis; LVOT indicates left ventricular outflow tract; VC, vena contracta; PHT, pressure half time; PISA, proximal isovelocity surface area; EROA, effective regurgitant orifice area; HRF, holodiastolic retrograde flow in the descending aorta; CMR, cardiovascular magnetic resonance imaging; RegV, regurgitant volume; TTE, transthoracic echocardiography; RegF, regurgitant fraction. * Statistically significant (*p* < 0.05). ^‡^ Primary MR patients were not included.

**Table 3:** Sex-specific association of LV size parameters per 1-SD increase (crude and adjusted for age) with presence of severe AR, defined as regurgitation fraction (RegF) ≥ 30% on cardiovascular magnetic resonance imaging (CMR).

LV Size Parameter	OR (95%CI) Per 1SD	*p*-Value	OR (95%CI) Per 1SD	*p*-Value
	Crude		Adjusted for Age	
MEN				
Echo LVEDD	3.80 [2.29–6.30]	<0.001	4.47 [2.61–7.66]	<0.001
Echo LVEDV	3.50 [2.12–5.53]	<0.001	4.26 [2.57–7.05]	<0.001
Echo LVEDV/BSA	3.93 [2.42–6.39]	<0.001	4.83 [2.81–8.31]	<0.001
CMR LVEDV	3.55 [2.20–5.73]	<0.001	3.74 [2.30–6.08]	<0.001
CMR LVEDV/BSA	4.04 [2.42–6.73]	<0.001	4.17 [2.49–6.97]	<0.001
Women				
Echo LVEDD	2.30 [1.33–3.98]	0.003	2.48 [1.40–4.38]	0.002
Echo LVEDV	2.12 [1.25–3.61]	0.005	2.26 [1.31–3.93]	0.004
Echo LVEDV/BSA	2.09 [1.23–3.53]	0.006	2.25 [1.30–3.89]	0.004
CMR LVEDV	2.63 [1.53–4.51]	<0.001	2.91 [1.65–5.14]	<0.001
CMR LVEDV/BSA	2.53 [1.49–4.28]	0.001	2.74 [1.57–4.80]	<0.001

LVEDD indicates left ventricular end-diastolic diameter; LVEDV, left ventricular end-diastolic volume; BSA, body surface area; CMR, cardiovascular magnetic resonance imaging.

**Table 4:** Univariable Cox regression composite of heart failure hospitalization, unscheduled AR intervention, and cardiovascular death.

	Crude HR (95% CI)	*p*-Value
Age	1.04 (1.03–1.60)	<0.001
Female Sex	1.69 (1.09–2.61)	0.018
Body Mass Index (kg/m^2^)	1.01 (0.96–1.06)	0.776
Hypertension	2.10 (1.26–3.49)	0.004
Atrial Fibrillation	1.82 (1.16–2.86)	0.009
Diabetes	1.86 (1.07–3.22)	0.027
Hyperlipidemia	1.56 (0.99–2.43)	0.051
Coronary Artery Disease	2.36 (1.50–3.69)	<0.001
Previous stroke	0.76 (0.19–3.11)	0.704
Hematocrit	0.91 (0.87–0.95)	<0.001
eGFR (mL/min/1.73 m^2^)	0.98 (0.97–0.99)	<0.001
Bicuspid aortic valve	0.12 (0.03–0.50)	0.003
CMR LVEDV/BSA (1-SD, mL/m^2^)	1.03 (0.812–1.30)	0.830
CMR RVEDV/BSA (1-SD, ml/m^2^)	1.11 (0.89–1.39)	0.353
CMR LVEF (%)	0.97 (0.95–0.99)	<0.001
CMR RVEF (%)	0.97 (0.96–0.99)	<0.001
CMR RegF (%)	1.02 (1.01–1.03)	0.004
CMR RegV (mL)	1.01 (0.99–1.02)	0.273

eGFR indicates estimated glomerular filtration rate; CMR, cardiovascular magnetic resonance imaging; RegF, Regurgitation Fraction; RegV, regurgitation volume.
